# Retraction: Up-Regulation of MiR-300 Promotes Proliferation and Invasion of Osteosarcoma by Targeting BRD7

**DOI:** 10.1371/journal.pone.0269904

**Published:** 2022-06-08

**Authors:** The PLOS ONE Editors

Following the publication of this article [1], similarities were noted between this article and articles submitted by other research groups, including [2–10], of which one article [10] was previously retracted [11].

Similarities included the following figures, which appear to fully or partially overlap, despite being published in different articles and representing different conditions:

- BRD7 panel in Fig 5D of [1] in grayscale, and the pCDNA-PAQR3 scamble panel in Fig 5E of [7].
- BRD7 panel in Fig 4C of [1], L1CAM panel in Fig 4D of [4], WASF3 panel in Fig 3D of [5], and PAQR3 panel in Fig 4D of [7].

The corresponding author did not comment on the above concerns. They provided data files which did not appear to contain the full raw data underlying the published figures. The concerns regarding the similarities between this article [1] and others [2–10] remain unresolved.

The unresolved concerns call into question the validity and provenance of the reported results, and the adherence of this article to the PLOS Authorship policy. Therefore, the *PLOS ONE* Editors retract this article [1].

In response to the retraction decision, all authors either did not respond directly or could not be reached.
